# Sputum Bacterial and Fungal Dynamics during Exacerbations of Severe COPD

**DOI:** 10.1371/journal.pone.0130736

**Published:** 2015-07-06

**Authors:** Jin Su, Hai-yue Liu, Xi-lan Tan, Yong Ji, Yun-xia Jiang, M Prabhakar, Zu-hua Rong, Hong-wei Zhou, Guo-xia Zhang

**Affiliations:** 1 State Key Laboratory of Organ Failure Research, Department of Environmental Health, Guangdong Provincial Key Laboratory of Tropical Disease Research, School of Public Health and Tropical Medicine, Southern Medical University, Guangzhou, China; 2 Department of Respiratory Physicians, Nanfang Hospital, Southern Medical University, Guangzhou, Guangdong, China; 3 Department of Hospital Infection Management, Zhujiang Hospital, Southern Medical University, Guangzhou, Guangdong, China; CAS, CHINA

## Abstract

The changes in the microbial community structure during acute exacerbations of severe chronic obstructive pulmonary disease (COPD) in hospitalized patients remain largely uncharacterized. Therefore, further studies focused on the temporal dynamics and structure of sputum microbial communities during acute exacerbation of COPD (AECOPD) would still be necessary. In our study, the use of molecular microbiological techniques provided insight into both fungal and bacterial diversities in AECOPD patients during hospitalization. In particular, we examined the structure and varieties of lung microbial community in 6 patients with severe AECOPD by amplifying 16S rRNA V4 hyper-variable and internal transcribed spacer (ITS) DNA regions using barcoded primers and the Illumina sequencing platform. Sequence analysis showed 261 bacterial genera representing 20 distinct phyla, with an average number of genera per patient of >157, indicating high diversity. *Acinetobacter*, *Prevotella*, *Neisseria*, *Rothia*, *Lactobacillus*, *Leptotrichia*, *Streptococcus*, *Veillonella*, and *Actinomyces* were the most commonly identified genera, and the average total sequencing number per sputum sample was >10000 18S ITS sequences. The fungal population was typically dominated by *Candia*, *Phialosimplex*, *Aspergillus*, *Penicillium*, *Cladosporium* and *Eutypella*. Our findings highlight that COPD patients have personalized structures and varieties in sputum microbial community during hospitalization periods.

## Introduction

Chronic obstructive pulmonary disease (COPD) is characterized by persistent limited airflow that is usually progressive and not fully reversible [1]. COPD is becoming a leading cause of mortality in China and imposes a heavy economic burden, with the total expenditure per patient amounting to 40% and ~33.3% of the average annual family income in urban and rural areas of China, respectively[2]. Acute exacerbation of COPD (AECOPD) [3] often develops into emergency situations that are associated with high morbidity and mortality, and regular treatment has no proven value during these emergencies [4]. A recent review noted that nearly half of AECOPD cases are due to bacterial infections [5]. AECOPD results in worsening of health status; limitation of activities; and an increase in comorbidities, including cardiovascular disease, osteoporosis and neuropsychiatric complications. Exacerbations also lead to a poorer prognosis by increasing the rate of disease progression and the annual decline in lung function [6].

Previous studies have demonstrated that in both healthy and diseased states, the respiratory tract is colonized by diverse communities of bacteria [7,8]. Due to mounting applications of high-throughput sequencing techniques, the number of studies on the microbial community of COPD patients is growing dramatically, and these studies have yielded exciting observations regarding the relationship between the lung microbiota and respiratory disease [1,7]. Many investigations have reported different structures of microbiota diversities between organs and between COPD patients [9,10]. The relationships between infection by specific microbial species and AECOPD as well as the relationships between COPD-associated pathogens and other microbes have informed our understanding of airway microbiology [11]. Many previous studies have focused on bacterial diversity, and very few have reported on fungal diversity [12,13]. Furthermore, it has been found that treatment with antibiotics alone primarily decreases the abundance of Proteobacteria and can result in prolonged suppression of certain microbial community members in patients with AECOPD [11]. Whereas the role of specific bacterial species in COPD has been extensively studied, other microbial species that may colonize the airways in COPD patients have not been analyzed. Therefore, the temporal dynamics of these communities and their responses to perturbations are not well understood. Until now, we could not clearly determine the treatment of AECOPD according to the lung microbiota composition. This issue deserves intense study to characterize the dynamics of the lung microbiota during the hospitalization of AECOPD patients.

As shown in a cohort study performed in 2014, the frequency of fungal infections in AECOPD patients peaks at 4.4% [14]. In particular, associations between *Aspergillus* colonization and COPD have been noted [15], and sensitization to *Aspergillus fumigatus* has been associated with reduced lung function in COPD patients. However, whether fungal colonization can serve as a marker of more severe lung disease and whether aggressive therapy is needed remain unknown [16]. Previous studies have shown that fungal infection is an important aspect of AECOPD that is worthy of intense research. Furthermore, colonization by viruses, *Chlamydophila* and fungi has become increasingly prevalent in AECOPD patients [17,18]. Nevertheless, few reports on the potential role of fungi in hospitalized AECOPD patients as well as on the special microbial community that these fungi are a part of have been published. Whether alteration in the fungal community will disturb the entire microbial structure and how it will further affect AECOPD thus remain to be studied.

We employed high-throughput sequencing of bacterial 16S rRNA V4 hyper-variable and fungal internal transcribed spacer (ITS) DNA regions to evaluate how the sputum microbiota of COPD patients changes each day during hospitalization. Our primary objective in the present study was to determine whether and, if so, how therapy influences the structure and composition of the bacterial and fungal communities in the sputum of COPD patients during hospitalization. Additionally, we sought to examine how the diversity of the microbial flora of sputum is changed by the presence of fungi.

## Materials and Methods

### Ethical Statement

This study was approved by the Ethical Committee of Southern Medical University. All participants provided written informed consent.

### Subjects

Patients experiencing an acute exacerbation of severe COPD and receiving medical treatment at Nanfang Hospital, Southern Medical University, were recruited for the study. All six subjects exhibited acute symptoms, such as cough, dyspnea, fatigue, and sputum production.

### Sample Collection

Sputum samples were obtained from six male individuals ranging in age from 73 to 83 years old, with an average age of 77 years. For a period of 7 to 16 days, each patient provided self-collected sputum samples until discharge from the hospital; the first 3 samples, which were collected on the first day, were collected before antibiotic consumption. All samples were obtained from June 2012 to September 2012 and were stored at -80°C until DNA extraction.

### DNA Extraction

DNA was extracted from the sputum of the patients as soon as the patients were diagnosed with severe COPD. After storage at -80°C, the sputum samples were thawed under ventilation for 20 min, and 2 μL from the inside portion of each sputum sample was placed in a 2-mL Eppendorf tube. Genomic DNA was extracted from each sputum sample using the Forensic Sample Nucleic Acid Extraction Kit (Bioeasy Technology, Inc., China) according to the manufacturer’s instructions.

### Bacterial 16S rRNA and Fungal ITS Gene Amplification

The 16S rRNA genes were amplified using barcoded V4 primers and were then purified and pooled as described by He et al. [19]. The ITS genes were amplified using barcoded ITSF: 5´ CTTGGTCATTTAGAGGAAGTAA 3´ and ITSR: 5´ GCTGCGTTCTTCATCGATGC 3´ primers. Each 20-μL reaction consisted of 10 μL of Maxima Hot Start PCR Master Mix (Thermo, USA), 2 μL of template DNA (approximately 100 ng), 0.5 μL of the barcoded ITSF primer (10 μM), 0.5 μL of the ITSR primer (10 μM), and 7 μL of nuclease-free water. The conditions for PCR included an initial hot-start incubation (15 min at 94°C), followed by 5 cycles of denaturation at 95°C for 30 s, annealing at 50°C for 30 s and extension at 72°C for 1 min; 35 cycles of denaturation at 95°C for 30 s, annealing at 65°C for 30 s and extension at 72°C for 1 min; and then a final extension at 72°C for 15 min. The 16S rRNA and ITS PCR products were sequenced at the Beijing Genomics Institution using paired-end sequencing with an Illumina HiSeq 2000 platform; 154 bp of the 16S rRNA and 70 bp of the ITS sequence were specifically sequenced from each end, with mismatches set at less than 10 bp, and used for later analysis. The sequences were deposited in the European Nucleotide Archive (ENA), and the accession numbers from ERS687383 to ERS687493.

### Sequence Processing and Analysis

The V4 hyper-variable region was sequenced by overlapping using the PE250 sequencing strategy. For quality-control purposes, we removed all of the sequences that contained ambiguous reads (N) and any sequences that contained mismatches in the forward or reverse primers. These clean, non-continuous sequences were then screened for chimeras using UCHIME [20]. A total of 3034133 high-quality sequencing reads of the 16S rRNA gene were generated after UCHIME use, 517850 of which belonged to fungi. The subsequent analyses were implemented using QIIME (1.5.0). Each 16S rRNA gene sample was normalized to 22000 sequences, and we used the non-normalized data to perform later fungal analyses. Before we analyzed the fungal data, we transformed the data using the constituent ratio. The sequences were then clustered using UCLUST, with a threshold distance set to 0.03, which corresponded to the species level. The Ribosome Database project (RDP) algorithm was applied to classify the representative sequences of each operational taxonomic unit (OTU) [21], the Shannon and PD_whole_tree indices were used to analyze alpha diversity, and PCoA analysis using QIIME based on the UniFrac distance was implemented [22]. The sequences were classified at the genus level and grouped into the next lowest taxonomic level. The sequences that were clearly fungal were used in an additional BLAST [23] analysis using the NCBI databank. Beta-diversity analyses were implemented based on the UniFrac distance, and all statistical analyses were performed using SigmaPlot 12.0.

## Results

### Patient Information

A total of 65 sputum samples were obtained from 6 subjects with severe COPD who had experienced one acute exacerbation. The clinical data of the patients were collected from reviews of each patient’s hospital medical records (S1 Table.). C-reactive protein (CRP) levels and the FEV1% were measured for all patients as a routine surveillance procedure at Nanfang Hospital, except for patient N3, for whom the FEV1% could not be measured because the patient did not have the power to release sufficient air for measurement.

### Microbial Diversity in the Sputum

**Fig 1:**
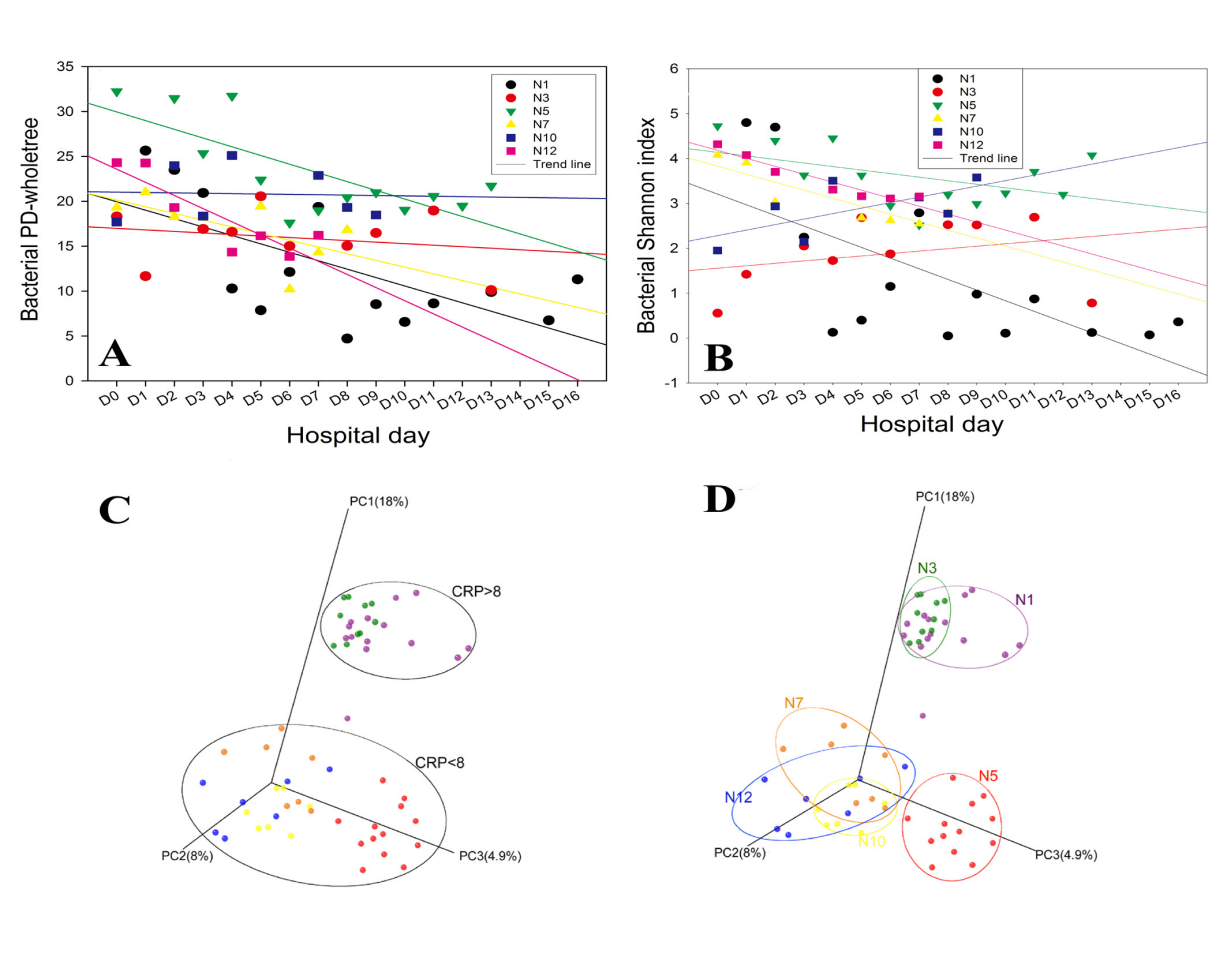
Distributions of the indices. (**A**) PD_whole_tree index, i.e., evenness index. (**B**) Shannon index, i.e., sample distribution index. (**C, D**) PCoA of unweighted UniFrac distance. (**C**) Community comparison of the 6 different subjects. (**D**) Two different groups based on the CRP value.

The sputum bacterial community structure varied among the different participants; Fig 1 shows the alpha- and beta-diversity results. We used the PD_whole_tree and Shannon indices to compare the alpha diversities of the different samples. There was considerable variability in the alpha diversity of each subject; however, no consistent patterns were observed. In subjects N1, N5, and N12, the PD_whole_tree and Shannon indices initially declined and then gradually increased over time. In subjects N3 and N10, there was an increase in the Shannon index throughout the observation period, whereas the PD_whole_tree index showed the opposite trend. For subject N7, the PD_whole_tree index showed a descending trend, with a mild increase over time, whereas the Shannon index showed a consistently descending trend. We additionally studied the PCoA of the UniFrac distances among the different subjects. The PCoA analysis of the sample distribution based on the unweighted UniFrac distance of each patient showed that the samples were separated, revealing that patients with severe COPD have a unique microbial community structure. These samples could be divided into two separate groups based on the CRP value.

**Fig 2:**
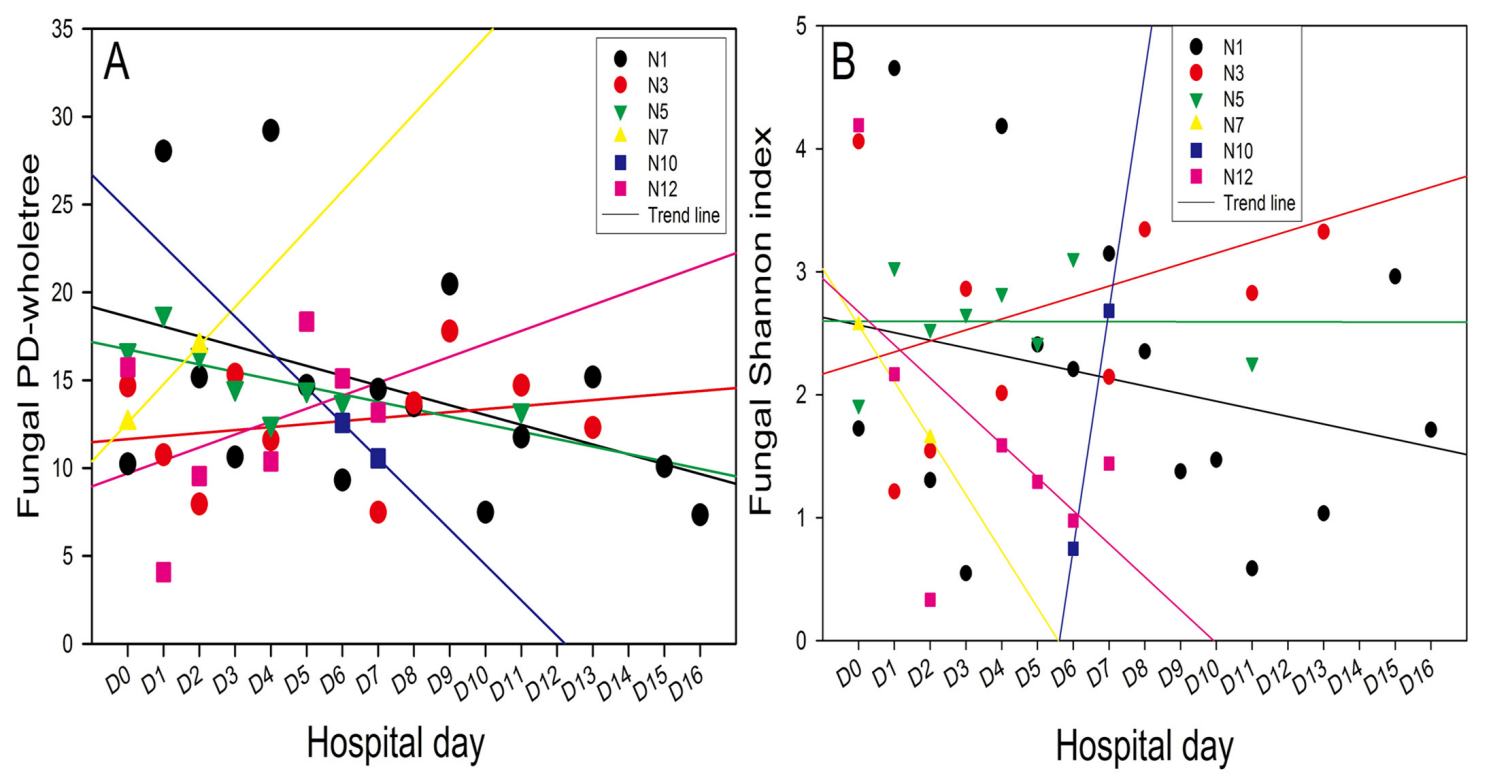
Distributions of the indices. (**A**) PD_whole_tree index, i.e., evenness index. (**B**) Shannon index of fungal diversity in the sputum samples of the 6 patients.

The fungal alpha-diversity analysis showed no consistent patterns (Fig 2), similar to the bacterial alpha-diversity results. The Shannon indices of patients N3 and N12 were higher on the first day, followed by a quick decrease and then a moderate increase at the end of the observation period. The opposite trend was observed for the N1 and N5 samples. Similar trends were also observed for the PD_whole_tree indices.

### Bacterial Communities

#### Phylum-level composition and temporal dynamics

**Fig 3:**
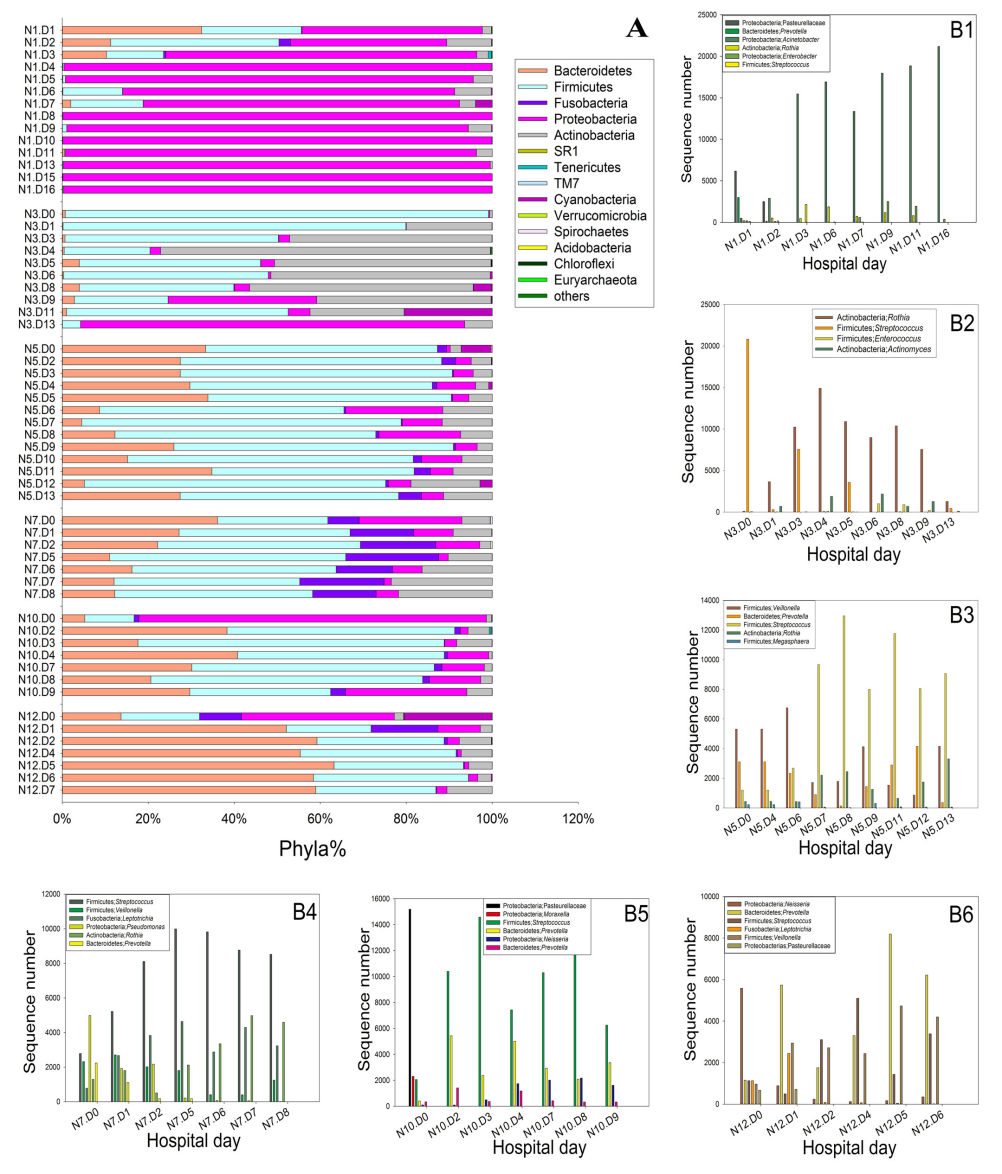
Comparison of the bacterial communities at the phylum level and of representative genera with highly abundant OTUs. (**A**) Taxonomic identification at the phylum level showing different phyla that varied with the period of hospitalization, the “other” represented for the phyla that did not being displayed directly. Patients with several significant changes in the OTU proportion over time: (**B1**) subject N1, (**B2**) subject N3, (**B3**) subject N5, (**B4**) subject N7, (**B5**) subject N10, and (**B6**) subject N12. The standardization of sequencing depth for each sample was 22000. N, case number; D, admission date.

Twenty phyla were detected in the sputum samples. Firmicutes (37.57%), Proteobacteria (29.12%), Bacteroidetes (17.91%), Actinobacteria (11.31%), Fusobacteria (2.93%) and Cyanobacteria (1.02%) were found at high percentages. In contrast, Spirochaetes, Euryarchaeota, Planctomycetes, Deinococcus-Thermus, Acidobacteria, Aquificae, Chlamydiae, Nitrospira, Chloroflexi, Crenarchaeota, Euryarchaeota, Synergistetes, SR1, Tenericutes and TM7 appeared at low percentages. Firmicutes and Proteobacteria were distributed widely and abundantly among all samples, and the percentage of Proteobacteria remained nearly stable, except in samples N10 and N12. Subject N10 had an increased percentage of *Neisseria* and a decreased percentage of *Moraxella*, whereas subject N12 had decreased percentages of *Neisseria* and *Moraxella*. Firmicutes were found at decreased percentages in subjects N1, N3, and N7, whereas subjects N5, N10, and N12 showed increased percentages; these results were attributed to changes in the abundance of the genus *Streptococcus*. The amount of Fusobacteria also changed significantly over time, and this result was reflected at all taxonomic levels, down to the genera *Leptotrichia* and *Fusobacterium*. *Streptophyta* was found to be the most abundant among members of the phylum Cyanobacteria, and *Rothia* and *Actinomyces* were the most unstable genera among members of the phylum Actinobacteria (Fig 3).

#### Highly abundant OTUs and their temporal dynamics in each patient.

Subjects with severe COPD showed unique microbial diversity at the genus level. *Streptococcus*, *Acinetobacter*, *Prevotella*, *Rothia*, *Veillonella*, *Pediococcus*, *Neisseria*, *Leptotrichia*, *Capnocytophaga*, *Porphyromonas*, and *Actinomyces* were regularly detected in all of the subjects (Table 1). Additionally, *Stenotrophomonas* was regularly detected in all subjects other than N12. Certain genera were highly abundant (>1% of their sequences) in several patients but did not regularly appear in other patients. For example, *Leptotrichia* regularly appeared only in subjects N5, N7, and N10; *Acinetobacter* was only highly abundant in subjects N1, N3 and N10; and *Actinomyces* was highly abundant in subjects N3 and N10.

**Table 1:** Genera accounting for more than 1% of the sequences for each subject.

N1	N3	N5	N7	N10	N12
*Acinetobacter*	*Acinetobacter*	*Campylobacter*	*Capnocytophaga*	*Acinetobacter*	*Capnocytophaga*
*Brevibacillus*	*Actinomyces*	*Capnocytophaga*	*Leptotrichia*	*Actinomyces*	*Corynebacterium*
*Enterobacter*	*Enterococcus*	*Lactobacillus*	*Neisseria*	*Capnocytophaga*	*Leptotrichia*
*Enterococcus*	*Lactobacillus*	*Leptotrichia*	*Porphyromonas*	*Leptotrichia*	*Neisseria*
*Prevotella*	*Pediococcus*	*Neisseria*	*Prevotella*	*Neisseria*	*Porphyromonas*
*Rothia*	*Rothia*	*Prevotella*	*Pseudomonas*	*Pediococcus*	*Prevotella*
	*Stenotrophomonas*	*Rothia*	*Rothia*	*Porphyromonas*	*Rothia*
	*Streptococcus*	*Streptococcus*	*Streptococcus*	*Prevotella*	*Streptococcus*
			*Veillonella*	*Rothia*	*Veillonella*
				*Stenotrophomonas*	
				*Streptococcus*	
				*Veillonella*	

In subject N1, the percentage of *Acinetobacter* increased throughout the observation period, the percentages of *Pasteurellaceae* and *Prevotella* quickly decreased over time, and the percentage of *Streptococcus* increased on the 2^nd^ and 3^rd^ days of the observation period and then decreased. The percentage of *Enterobacter* remained stable until day 9, after which it increased until the end of the observation period. *Rothia* and *Neisseria* were stable throughout the observation period. In subject N3, the percentage of *Rothia* increased quickly on the 2^nd^ day, whereas the percentage of *Streptococcus* decreased, and the percentages of *Actinomyces* and *Acinetobacter* increased until the 6^th^ day and then decreased. In subject N5, the percentage of *Prevotella* decreased until the 9^th^ day and then quickly increased, and those of *Streptococcus* and *Rothia* increased until the end of the observation period. In subject N7, the percentages of *Streptococcus* and *Leptotrichia* increased before the 6^th^ day and then slightly decreased, the percentage of *Veillonella* showed the completely opposite trend, the percentages of *Pseudomonas* and *Prevotella* quickly decreased starting on day 1, and the percentage of *Rothia* decreased slightly during the first two days and then increased. In subject N10, the percentages of *Pasteurellaceae* and *Moraxella* decreased beginning on day 1, the percentages of *Streptococcus* and *Prevotella* increased, and the percentage of *Neisseria* increased slightly starting on the 2^nd^ day. In subject N12, *Neisseria*, *Leptotrichia* and *Pasteurellaceae* nearly disappeared starting on the first day, whereas the percentages of *Prevotella*, *Streptococcus* and *Veillonella* rose steadily, except on the 4^th^ and 5^th^ days, when they decreased slightly.

#### Temporal dynamics of fungal communities

**Fig 4:**
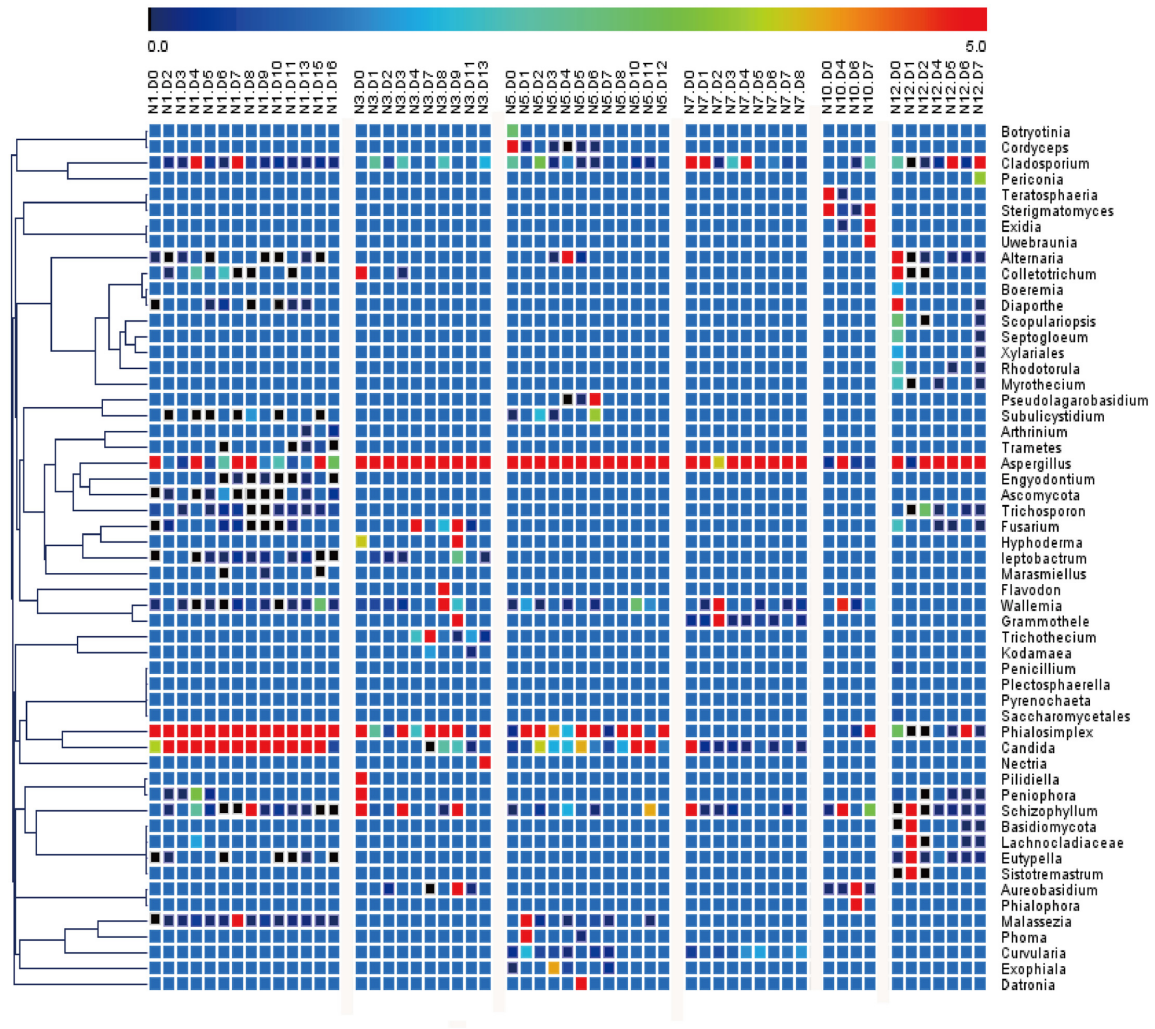
System clustering of fungal composition at the genus level. The names of several of the most abundant genera that included the terminal taxa shown in the heatmap are listed on the right of the figure. The sample names are listed at the top of the heatmap. “N” represents samples from different subjects, and the color bar at the top of the heatmap shows the percentages of <5% of the sequences in each sputum sample.

The fungal community in the lung microbiome of each patient was analyzed by ITS gene sequencing, and system clustering was performed to obtain a visual representation of the overall similarity among the sputum samples. Fig 4 shows the relative abundance and system clustering trees of the samples based on the percentage of fungal operational units in each community. Each patient exhibited an unstable microbial community during the observation period. The lung microbiomes of subjects N5, N7, and N12 were dominated by *Aspergillus*, whereas the other two subjects had intermediate or low levels of *Aspergillus*. The microbiome of subject N1 was dominated by *Phialosimplex* and *Candida*, whereas the microbiome of subject N3 was dominated by *Aspergillus* and *Phialosimplex*. The microbiome of subject N10 was dominated by *Teratosphaeria* and *Sterigmatomyces* after the patient entered the hospital and then by *Aureobasidium* afterward.

## Discussion

In this study, we analyzed variations on the bacteria and fungi in the sputum of patients hospitalized with severe COPD. In contrast to most previous studies, which described particular community compositions during exacerbation [10,24], the vertical sampling employed in our experimental design allowed us to more closely follow the day-to-day changes in sputum microbiota during hospital stays.

Several changes were common, i.e., the amounts of Firmicutes, Proteobacteria and Fusobacteria changed significantly over time, and these changes were noted at all taxonomic levels, down to the genera *Streptococcus*, *Lactobacillus*, *Veillonella*, *Moraxella*, *Neisseria*, *Leptotrichia* and *Fusobacterium*. Changes in the phyla Actinobacteria and Bacteroidetes were also reflected by changes in the genera *Rothia*, *Corynebacterium*, *Actinomyces*, *Capnocytophaga* and *Prevotella*.

About 50% of COPD exacerbations involve bacteria, with the most frequently isolated species being *Haemophilus influenzae*, *Pseudomonas aeruginosa*, *Moraxella catarrhalis*, *Streptococcus pneumoniae*, *Staphylococcus aureus*, *Haemophilus haemolyticus*, and *Haemophilus parainfluenzae* [25]. Our results showed that the dynamic patterns of change in sputum microbial communities showed significant variation among patients, and these patterns were almost always personalized to each patient. Most of the patients experienced some changes in community composition during their hospital stay; however, no general trends were apparent.

In our research, a shift in the relative abundance of a few populations was noted; however, no single population became dominant. Although the sampling locations were different, the same results were found in a study by Sze et al. [10]. The results of our bacterial community composition analysis (Fig 2) also agreed with findings reported by Huang, who stated that individuals with greater bacterial community diversity also supported a large number of Firmicutes and that those with less bacterial diversity had a greater number of Proteobacteria [26]. Our results showed that *Leptotrichia* and *Streptococcus* are the main genera associated with Fusobacteria and Firmicutes, respectively, in severe COPD patients. These findings have got some differences with the previous research by Sze, whose research indicating that 7 in 8 samples, *Lactobacillus* is the main genus associated with an increased amount of Firmicutes, in one sample, *Streptococcus* being the main genus associated with an increased amount of Firmicutes [10]. The *Aspergillus* genus was found in the sputum samples of all six patients. In a previous study, a positive *Aspergillus* culture was reported to affect grade 3 or 4 COPD [27].

Our study focused on severe COPD and revealed that the most common bacterial genera found in COPD patients were *Acinetobacter*, *Prevotella*, *Neisseria*, *Rothia*, *Lactobacillus*, *Leptotrichia*, *Streptococcus*, *Veillonella*, *Pasteurella*, *Klebsiella* and *Actinomyces*. Among these, *Streptococcus*, *Prevotella* and *Neisseria* have also been found in healthy subjects [28]. Our data clearly disagree with the hypothesis that the bronchial microbiomes of COPD patients contain an increased presence of *Haemophilus*, *Moraxella* and *Pseudomonas* spp. [29–31]. Sethi et al. found that the presence of *H*. *influenza* and *M*. *catarrhalis* was associated with increased inflammatory markers in the sputum, but this finding was not supported by the data in the present study because subject N1, who had the highest CRP level, had negligible abundances of *H*. *influenza* and *M*. *catarrhalis* [32]. The microbiomes clustered into two different regions based on the level of CRP, as clearly demonstrated by unweighted UniFrac analysis. Similar research was performed by Clark et al., who found that CRP levels are closely associated with the virus and mixed virus/bacteria detection rates in COPD patients [33]. Additionally, CRP has been reported to steadily increase the effects of antibiotic treatments [34].

To our knowledge, we are the first to combine bacterial 16S rRNA and fungal ITS sequence analyses to characterize sputum microbiota in severe COPD patients and to analyze samples from each day of hospitalization during COPD exacerbation. The importance of daily dynamics research into how bacterial and fungal communities vary among AECOPD patients was unknown until now. Several important studies previously collected samples from severe COPD patients, but they were limited in their ability to characterize the bacteria that were present in these samples. Nonetheless, there are certain limitations to our study that impede us from drawing broad conclusions. The number of individuals enrolled in this preliminary study was small, which limited our ability to conduct rigorous statistical analyses. Additionally, the cultivation-independent methods employed here could not produce estimates of the quantitative changes observed in previous studies. Furthermore, we suspect that many factors may influence the dynamics of sputum communities during exacerbations, such as the types of antibiotics used, complications, and elements of the host immune system, the level of CRP, the mucosal environment, and routine monitoring indexes, among others. To assess the importance of these variables in sputum community dynamics, larger studies that replicate treatments over consecutive exacerbation periods will be necessary. Additionally, whether the specific compositional or potential biomarker genera that were detected in the airway microbiome could be useful indicators of exacerbation remains to be determined in further studies.

## Conclusion

In summary, this study has indicated that the temporal dynamics and diversity of sputum microbial community of 6 very several COPD patients during hospitalization period. The structure and the varieties of sputum microbial community were personalized and the structures of sputum microbial composition have changed rapidly every day during hospitalization period. The bacteria population was typically represented by *Acinetobacter*, *Prevotella*, *Neisseria*, *Rothia*, *Lactobacillus*, *Leptotrichia*, *Streptococcus*, *Veillonella*, and *Actinomyces*. The fungal population was typically dominated by *Candia*, *Phialosimplex*, *Aspergillus*, *Penicillium*, *Cladosporium* and *Eutypella*. Among the entire genus there have got some genus that changed rapidly and enormously in the abundance of the sputum microbial community which were *Pseudomonas*, *Streptococcus*, *Moraxella*, *Neisseria*, *Veillonella* and *Aspergillus*.

## Supporting Information

S1 Table Clinical features of and microbiological findings from 6 severe AECOPD patients. (DOCX)
