# Supplementary material for: Sputum Bacterial and Fungal Dynamics during Exacerbations of Severe COPD
Source: PLoS One. 2015 Jul 6;10(7):e0130736. doi: 10.1371/journal.pone.0130736 (PMC4493005; doi:10.1371/journal.pone.0130736)
Supplement: S1 Table — (DOCX) [file pone.0130736.s001.docx]

| **Patient** | **Bacterial culture** | **Fungal culture** | **Medication** | **CRP** | **FEV1/FVC** | **FEV1%** | **Age** | **Gender** | **Hospital stay (days)** |
| --- | --- | --- | --- | --- | --- | --- | --- | --- | --- |
| **N1** | ***Acinetobacter baumannii*** | ***Candida albicans*** | **Cst+Etimicin** | **95.3** | **51.66** | **57.5** | **83** | **Male** | **16** |
| **N3** | **Normal** | ***Candida tropicalis*** | **Meropenem** | **9.8** | **Nd** | **None** | **80** | **Male** | **13** |
| **N5** | **Normal** | **None** | **Cst** | **0.3** | **39.14** | **31.2** | **79** | **Male** | **8** |
| **N7** | **Normal** | **None** | **Cst+Teicoplanin** | **3.6** | **68.22** | **26.2** | **76** | **Male** | **8** |
| **N10** | **None** | **none** | **Cst** | **2** | **41.90** | **64.62** | **71** | **Male** | **11** |
| **N12** | **Normal** | **None** | **Cst+Etimicin** | **0.4** | **None** | **None** | **73** | **Male** | **7** |

Definitions of abbreviations: Cst = cefoperazone sodium and tazobactam; Nd, not detected
